# Gender differences in CNV burden do not confound schizophrenia CNV associations

**DOI:** 10.1038/srep25986

**Published:** 2016-05-17

**Authors:** Jun Han, James T. R. Walters, George Kirov, Andrew Pocklington, Valentina Escott-Price, Michael J. Owen, Peter Holmans, Michael C. O’Donovan, Elliott Rees

**Affiliations:** 1 Medical Research Council Centre for Neuropsychiatric Genetics and Genomics, Cardiff University, Cardiff, Wales, UK; 2 Division of Psychological Medicine and Clinical Neurosciences, Cardiff University, Cardiff, Wales, UK

## Abstract

Compared with the general population, an excess of rare copy number variants (CNVs) has been identified in people with schizophrenia. Females with neurodevelopmental disorders and in the general population have been reported to carry more large, rare CNVs than males. Given that many schizophrenia datasets do not have equal gender ratios in cases and controls, sex differences in CNV burden might have impacted on estimates of case-related CNV burden and also on associations to individual loci. In a sample of 13,276 cases and 17,863 controls, we observed a small but significant excess of large (≥500 Kb), rare (<1%) CNVs in females compared with males in both cases and controls (OR = 1.17, P = 0.0012 for controls; OR = 1.11, P = 0.045 for cases). The burden of 11 schizophrenia-associated CNVs was significantly higher in female cases compared with male cases (OR = 1.38, P = 0.0055), but after accounting for the rates of CNVs in controls, we found no significant gender difference in the risk conferred by these loci. Controlling for gender had a negligible effect on the significance of association between specific CNVs and schizophrenia. The female excess of large CNVs in both cases and controls suggests a female protective mechanism exists for deleterious CNVs that may extend beyond neurodevelopmental phenotypes.

Schizophrenia has a strong genetic component involving rare and common alleles distributed across many genes^1^,^2^. Common alleles confer weak effects (OR < 1.1 in general) but collectively account for a substantial proportion of genetic liability to the disorder^3^. On the other hand, some rare genetic variants, such as copy number variants (CNVs), confer larger risks for schizophrenia^4^,^5^. A combined analysis of published datasets has shown strong evidence for association between 11 individual CNVs and risk of schizophrenia and weaker evidence for 4 additional risk CNVs^6^. Duplications of 22q11.2 have also been associated with reduced risk of schizophrenia^7^.

A gender-bias in prevalence has been repeatedly observed in neurodevelopmental disorders. In autism spectrum disorder (ASD), the mean male-to-female ratio is about 4:1^8^, whereas in schizophrenia the male-to-female ratio is 1.4:1, though this varies by age reflecting sex related differences in age at onset^9^. Sex differences in schizophrenia have also been reported for structural and functional brain abnormalities whose origins are thought to occur during brain development^10^,^11^,^12^,^13^,^14^.

A recent study found an excess of deleterious autosomal CNVs in females compared with males in a neurodevelopmental disorders cohort and in an ASD cohort^15^. Females with ASD have also been found to possess a higher burden of *de novo* CNVs and genes disrupted by *de novo* CNVs than males with ASD^16^,^17^,^18^. The lower prevalence of ASD and higher mutational burden in females with the disorder is consistent with a different liability threshold for females compared to males whereby females require a greater risk factor load to manifest neurodevelopmental disorders^15^,^19^,^20^. A higher female autosomal burden of large, rare CNVs, attributed to lower rates of foetal loss, has also been reported in the general population^21^.

Given the excess burdens of CNVs in females in both neurodevelopmental and control samples, we tested whether similar differences in burden exist in schizophrenia using a large case control dataset (N = 31,139 individuals). We evaluated the CNV burden in females and males at a genome-wide level and for 11 strongly associated schizophrenia risk loci. We also tested whether association between specific CNVs and schizophrenia are robust to adjusting for gender. Consistent with earlier findings, we found an increased CNV burden for large (≥500 Kb), rare (<1%) CNVs in female controls and now report an excess in female cases with the disorder. Although female cases also had an excess burden compared with males for 11 CNVs that have been implicated in schizophrenia, the association between those CNVs and schizophrenia was not diminished after controlling for gender.

## Results

### Females carry an excess of CNVs both genome-wide and at specific loci

**Figure 1:**
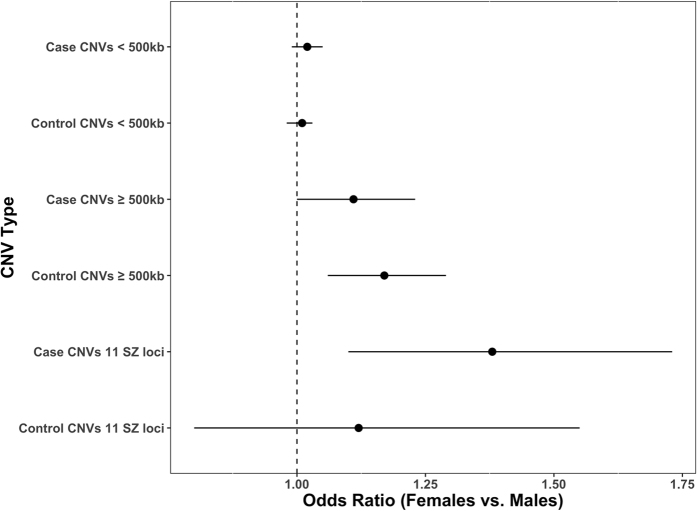
Gender CNV burden in the combined dataset. CNV burden is compared between males and females for both case and control samples. The combined CLOZUK, MGS and ISC dataset consists of 9,172 male and 4,104 female case samples, and 8,807 male and 9,056 female control samples.

Females had a genome-wide excess of large (≥500 kb) and rare (<1%) CNVs in cases (OR = 1.11, 95% CI = 1.00–1.23, P = 0.045) and controls (OR = 1.17, 95% CI = 1.06–1.29, P = 0.0012) (Fig. 1, Supplementary Table S1) although only in female controls would this survive correction (P = 0.0048) for 4 independent tests (CNVs < 500kb and CNV ≥ 500 kb separately for cases and controls). While the excess CNV burden in female cases does not survive correction for multiple testing, importantly the size of the effect is not significantly different from that in female controls (Z-test P = 0.24). The effect size for schizophrenia conferred by all large, rare CNVs in females (OR = 1.24, 95% CI = 1.12–1.38, P = 8.22 × 10^−5^) and in males (OR = 1.32, 95% CI = 1.20–1.45, P = 4.13 × 10^−9^) did not statistically differ (Z-test P = 0.19).

When deletions and duplications were analysed separately (Supplementary Table S1), we found that the contribution from large deletions to the excess of CNVs in control females was significantly greater than that from large duplications (Z-test P = 0.013). In cases, no significant difference was observed between the contribution of large deletions and duplications to the CNV excess in females (Z-test P = 0.44, Supplementary Table S1). No difference in the burden of smaller CNVs (<500 kb) was observed between males and females for cases or controls (Fig. 1, Supplementary Table S1). A breakdown of CNV burden in the datasets that constitute our case and control samples can be found in Supplementary Table S2. Despite the excess burden of large deletions in female controls, we found no significant difference between males and females for the number of genes overlapping CNVs for any class of CNV tested (Supplementary Table S3).

The burden of 11 schizophrenia risk CNVs was significantly higher in female cases than male cases (OR = 1.38, 95% CI = 1.10–1.73, P = 0.0055) but not in female controls compared with male controls (OR = 1.12, 95% CI = 0.80–1.55, P = 0.52) (Fig. 1, Supplementary Table S1). However, the difference in effect size between these two tests did not significantly differ (Z-test P = 0.15). When we compared the risk for schizophrenia conferred by the set of 11 CNV loci in females (OR = 3.56, 95% CI = 2.66–4.75, P = 2.06 × 10^−18^) and males (OR = 2.85, 95% CI = 2.15–3.76, P = 3.66 × 10^−15^), we found no significant difference in effect size (Z test P = 0.14). Hence, the non-significant test by sex in the controls might reflect lower power given that (by definition) there are fewer observations of these pathogenic CNVs in controls.

### Association of specific CNVs with schizophrenia is not confounded by gender

Table 1 shows association statistics between individual CNVs and schizophrenia. These CNVs include 11 known schizophrenia risk loci, 4 additional risk CNVs that have yet to be implicated in schizophrenia with strong evidence, and 1 protective CNV (see methods for detail). Controlling for gender had a negligible effect on the significance of association between specific CNVs and schizophrenia. Table S4 presents a breakdown of these associations for the individual datasets (CLOZUK, ISC and MGS).

## Discussion

We have carried out an analysis of gender stratified CNV burden in schizophrenia at both a genome-wide level and for previously associated loci. We restricted our analysis to the autosome as sex chromosomal CNVs were not available for all samples. We found a genome-wide excess of large, rare CNVs in females compared with males, irrespective of disease status. Similar observations have been reported in ASD and general population cohorts^15^,^21^. We also report an excess burden of CNVs at 11 schizophrenia risk loci in female cases compared with male cases, an observation that is reminiscent of an excess of deleterious variants in females with childhood neurodevelopmental disorders^15^. However, when males and females were analysed separately, we found no difference in effect size for risk of schizophrenia conferred by the total burden of the 11 associated loci. Moreover, association of individual CNV loci was not affected when statistical tests were used that accounted for gender. These results confirm that associations between schizophrenia and 11 specific risk CNVs and the protective 22q11.2 duplication are robust to differences in gender proportion in case and control samples. The weaker evidence for 4 additional risk CNVs cannot be attributed to gender related heterogeneity. Interestingly, despite the excess number of CNVs in females, we found no significant difference by sex in the number of genes disrupted by CNVs, suggesting that on average, CNVs are less gene-dense in females than they are in males.

At a genome-wide level, an excess female CNV burden was only observed for large CNVs (≥500 kb). However, we cannot confidently exclude a similar effect from smaller CNVs given the limited resolution to which microarrays can accurately call CNVs. When deletions and duplications ≥500 Kb were analysed separately, we found that deletions primarily accounted for the excess genome-wide CNV burden seen in female controls.

Recent findings of an excess mutational burden in females provide support for the view that females are relatively protected from neurodevelopmental disorders^15^,^17^,^18^,^19^,^22^,^23^. Schizophrenia is now generally considered to be at least in part a neurodevelopmental disorder; it is known to share genetic risk alleles with autism and other neurodevelopmental disorders^24^,^25^ and there is also an excess risk of the disorder in males, albeit much more modest than it is for other neurodevelopmental disorders such as ID, ASD and ADHD. The excess burden of CNVs in females might be considered evidence for female robustness to schizophrenia but caution is required given the pattern of findings in controls. As we and others^21^ have shown, female controls also have higher rates of large, rare CNVs, which in general are the class of CNV that contribute to neurodevelopmental disorders. This elevation in rate of CNVs in both cases and controls could potentially point to a mechanism that goes beyond protection from disorders such as ASD and schizophrenia: for example, protection from female foetal loss or premature death (pre- or post-natal). However, to address this question requires truly representative population cohorts as the gender CNV bias in controls might be an artefact resulting from female protection from conditions (including neurodevelopmental disorders) that would normally result in exclusion from control cohorts.

It is clear that CNV burden differs between males and females in both patients diagnosed with neurodevelopmental disorders, such as schizophrenia, and also in the general population. However, our data indicate that variation in gender CNV burden does not impact on known schizophrenia-CNV associations. We provide further evidence that in both case and control samples, the rate of deleterious CNVs is greater in females than in males, although investigating the reason for this will require large population cohorts.

## Methods

### Study samples and QC

Case and control CNVs were derived from three published samples: CLOZUK^6^, the ISC^4^ and the MGS^26^. A full description of these samples, the arrays they were genotyped on, and CNV calling procedures can be found in the original publications^4^,^6^,^26^. Quality control was performed to remove low quality samples (see original publications), and details of those samples that passed quality control in each study can be found elsewhere^6^. We excluded samples of unknown gender (sex chromosome molecular genetic data were not available for all samples) and putative CNVs < 15 kb in size and/or covered by <15 probes. After filtering, a total of 31,139 individuals were included in the current study (17,959 from CLOZUK and its corresponding controls, 6,600 from MGS and 6,580 from the ISC, Table 2). All CNVs have a population frequency <1%.

### Selection of specific schizophrenia-associated CNVs

We tested the burden of 11 schizophrenia risk CNV loci that showed significant association with schizophrenia in our previous publication^6^; deletions at 1q21.1, *NRXN1* (exonic CNVs only), 3q29, 15q11.2, 15q13.3 and 22q11.2; duplications at 1q21.1, 16p11.2 and 16p13.11, the Prader-Willi/Angelman syndrome (PWS/AS) region and Williams-Beuren syndrome (WBS) region. In our analysis of individual CNV loci, we tested these 11 schizophrenia risk CNVs, along with previously implicated risk CNVs for which the overall evidence is less strong^6^, postulating some of the variable evidence here might be due to gender related heterogeneity. These included duplications of *VIPR2*, deletions of 17p12, 17q12 and distal 16p11.2. We also tested the impact of gender on protective effects for 22q11.2 duplication^7^.

### Statistical analysis

We tested for CNV burden (≥500 kb; <500 kb) and burden of 11 schizophrenia risk loci in females versus males by comparing the change in deviance between the following logistic regression models (R glm function, family = binomial(“logit”)) using a two-sided test (ANOVA). Odds ratios (ORs) were derived from the correlation coefficients for number of CNVs.

1. * logit* (*pr* (*gender*)) ~ *number of CNVs* + *study source* + *microarray platform*
2. * logit* (*pr* (*gender*)) ~ *study source* + *microarray platform*

To test whether females had more genes disrupted by CNVs than males, a similar analysis was performed where ‘number of CNVs’ in the above model was replaced with ‘number of genes overlapping CNVs’.

To test whether the risk of schizophrenia conferred by large CNVs (≥500 kb) or the set of 11 specific schizophrenia CNVs is significantly different for females and males, we compared the effect size in females with the effect size in males using the coefficients and standard errors (Z-test, see below) derived from the following logistic regression model (R glm function, family = binomial(“logit”)):

*logit* (*pr*(*case*)) *~ number of schizophrenia CNVs* + *study source* + *microarray platform*

We note that almost identical P values were obtained when the above model was replaced with Firth’s procedure^27^ (data not shown).

Differences in effect size between tests were evaluated with a Z-test, which was constructed using the equation (1)


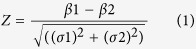


where *β* is the coefficient for the variable of interest taken from the regression model and σ is that variable’s standard error. P values were generated by comparing the absolute of the Z statistic against the cumulative distribution function of the standard normal distribution.

To assess whether a difference in gender proportion between cases and controls confounds association between schizophrenia and specific CNVs, we calculated individual locus association statistics using a Fisher’s Exact test (two-sided) and a Cochran-Mantel-Haenszel Exact test (two-sided) that was stratified by gender.

All statistical tests were conducted using R Statistical Software (https://www.r-project.org).

## Additional Information

**How to cite this article**: Han, J. *et al*. Gender differences in CNV burden do not confound schizophrenia CNV associations. *Sci. Rep.* **6**, 25986; doi: 10.1038/srep25986 (2016).

## Supplementary Material

Supplementary Information

Supplementary Information

## Figures and Tables

**Table 1:** Effect of gender stratification on schizophrenia CNV associations.

Locus	N_CNV_ in cases (Frequency, % in males/% in females)	N_CNV_ in controls (Frequency, % in males/% in females)	SCZ association		SCZ association controlled for gender	
			Odds Ratio (95% CI)	P-value (two-sided Fisher’s Exact test)	Odds Ratio (95% CI)	P-value (two-sided Cochran-Mantel-Haenszel Exact test)
15q11.2 del§	84 (0.57/0.78)	73 (0.43/0.39)	1.55 (1.12–2.16)	0.0074	1.59 (1.14–2.22)	0.0050
15q13.3 del§	17 (0.14/0.10)	4 (0.01/0.03)	5.73 (1.87–23.39)	0.00053	5.66 (1.81–23.47)	0.00062
16p11.2 distal del*	5 (0.02/0.07)	4 (0.01/0.03)	1.68 (0.36–8.48)	0.51	2.12 (0.44–10.95)	0.30
16p11.2 dup§	42 (0.31/0.34)	5 (0.02/0.03)	11.33 (4.49–36.68)	3.29 × 10^−11^	11.66 (4.57–38.08)	7.11 × 10^−11^
16p13.11 dup§	42 (0.25/0.46)	40 (0.19/0.25)	1.41 (0.89–2.24)	0.12	1.55 (0.97–2.48)	0.065
17p12 del*	9 (0.03/0.15)	7 (0.01/0.07)	1.73 (0.57–5.47)	0.32	2.33 (0.76–7.50)	0.11
17q12 del*	2 (0.00/0.05)	0 (0.00/0.00)	NA (0.25–∞)	0.18	NA (0.41–∞)	0.097
1q21.1 del§	24 (0.16/0.22)	6 (0.03/0.03)	5.39 (2.15–16.13)	4.80 × 10^−5^	5.64 (2.21–17.10)	3.77 × 10^−5^
1q21.1 dup§	16 (0.10/0.17)	8 (0.02/0.07)	2.69 (1.09–7.27)	0.022	3.11 (1.23–8.53)	0.0097
3q29 del§	10 (0.05/0.12)	0 (0.00/0.00)	NA (3.02–∞)	0.00020	NA (3.46–∞)	0.00010
22q11.2 del§	48 (0.32/0.46)	0 (0.00/0.00)	NA (16.90–∞)	1.61 × 10^−18^	NA (18.14–∞)	7.70 × 10^−19^
22q11.2 dup^	2 (0.01/0.02)	17 (0.07/0.12)	0.15 (0.02–0.67)	0.0043	0.18 (0.02–0.77)	0.014
*NRXN1* del§	24 (0.16/0.22)	5 (0.05/0.01)	6.47 (2.42–21.71)	1.34 × 10^−5^	6.48 (2.38–22.02)	2.57 × 10^−5^
PWS/AS dup§	11 (0.09/0.08)	0 (0.00/0.00)	NA (3.38–∞)	8.44 × 10^−5^	NA (3.36–∞)	8.49 × 10^−5^
*VIPR2* dup*	12 (0.11/0.05)	14 (0.07/0.09)	1.15 (0.49–2.69)	0.84	1.12 (0.46–2.67)	0.84
WBS dup§	6 (0.05/0.04)	2 (0.00/0.02)	4.04 (0.72–40.93)	0.080	5.10 (0.94–51.62)	0.037

Association between schizophrenia and CNVs at 15 specific risk loci and the protective 22q11.2 duplication locus. Association statistics are calculated with a Fisher’s Exact test (two-sided) and a Cochran-Mantel-Haenszel Exact test (two-sided) stratified by gender. SCZ = schizophrenia, PWS/AS = Prader-Willi/Angelman syndrome. WBS = Williams-Beuren syndrome.

^§^Risk locus (strong evidence).

^*^Risk locus (weaker evidence).

^Protective locus.

**Table 2:** Female and male samples included in the schizophrenia datasets.

Datasets	Cases			Controls		
	N_males_	N_females_	N_males_/N_females_	N_males_	N_females_	N_males_/N_females_
CLOZUK	4,766	1,938	2.46	5,762	5,493	1.05
MGS	2,162	1,015	2.13	1,567	1,856	0.84
ISC	2,244	1,151	1.95	1,478	1,707	0.87
Total	9,172	4,104	2.23	8,807	9,056	0.97
