# Supplementary material for: Gender differences in CNV burden do not confound schizophrenia CNV associations
Source: Sci Rep. 2016 May 17;6:25986. doi: 10.1038/srep25986 (PMC4869015; doi:10.1038/srep25986)
Supplement: Supplementary Information [file srep25986-s1.pdf]

Supplementary Material

Gender differences in CNV burden do not confound schizophrenia CNV associations

Jun Han[1], James T.R. Walters[1], George Kirov[1], Andrew Pocklington[1], Valentina Escott-Price[1], Michael J Owen[1,2], Peter Holmans[1], Michael C O'Donovan[1], Elliott Rees[1]

Affiliations

1. Medical Research Council Centre for Neuropsychiatric Genetics and Genomics, Cardiff University, Cardiff, Wales, UK
2. Division of Psychological Medicine and Clinical Neurosciences, Cardiff University, Cardiff, Wales, UK

Table contents

1. Gender CNV burden in the combined dataset
2. Breakdown of the gender CNV burden observed in the different datasets that comprise our main analysis
3 Gender burden of CNV gene content in combined dataset
4. Gender stratification effect on schizophrenia CNV associations in the different datasets that comprise our main analysis

1. Gender CNV burden in the combined dataset

Table S1. Gender CNV burden in combined dataset

CNV Type	Cases§				Controls§			
	Male rate (N CNVs)	Female rate (N CNVs)	OR (95% CI)*	P-value^	Male rate (N CNVs)	Female rate (N CNVs)	OR (95% CI)*	P-value^
**All < 500kb**	1.51 (13,826)	1.63 (6,674)	1.02 (0.99-1.05)	0.19	1.33 (11,734)	1.44 (13,075)	1.01 (0.98-1.03)	0.69
**Del < 500kb**	0.78 (7,125)	0.85 (3,485)	1.03 (0.99-1.07)	0.19	0.68 (5,984)	0.76 (6,866)	1.02 (0.99-1.06)	0.20
**Dup < 500kb**	0.73 (6,701)	0.78 (3,189)	1.01 (0.97-1.05)	0.56	0.65 (5,750)	0.69 (6,209)	0.99 (0.95-1.02)	0.47
**All ≥ 500kb**	0.12 (1,095)	0.14 (553)	**1.11 (1.00-1.23)**	**0.045**	0.088 (774)	0.11 (961)	**1.17 (1.06-1.29)**	**0.0012**
**Del ≥ 500kb**	0.035 (324)	0.041 (167)	1.12 (0.93-1.35)	0.22	0.018 (156)	0.026 (238)	**1.43 (1.17-1.74)**	**0.00043**
**Dup ≥ 500kb**	0.084 (771)	0.094 (386)	1.10 (0.98-1.25)	0.11	0.070 (618)	0.08 (723)	1.10 (0.99-1.23)	0.078
**11 Loci**	0.022 (200)	0.030 (124)	**1.38 (1.10-1.73)**	**0.0055**	0.0076 (67)	0.0084 (76)	1.12 (0.80-1.55)	0.52

§ Combined CLOZUK, MGS and ISC dataset contains 9,172 males and 4,104 females in case samples, and 8,807 males and 9,056 females in control samples.
*Odds ratios (and 95% confidence intervals) of females *vs* males for the number of CNVs they carry.
^P-values of correlation of number of CNVs and gender generated from generalised linear logistic regression model.
All: deletions and duplications
Del: deletions
Dup: duplications

2. Breakdown of the gender CNV burden observed in the different datasets that comprise our main analysis

Table S2. Gender CNV burden in CLOZUK, MGS and ISC datasets

Dataset	CNV Type	Cases§				Controls§			
		Male (N CNVs)	Female (N CNVs)	OR (95% CI)*	P-value^	Male (N CNVs)	Female (N CNVs)	OR (95% CI)*	P-value^
CLOZUK	**All < 500kb**	4476	1826	1.00 (0.95-1.06)	0.91	5133	4945	1.01 (0.97-1.05)	0.61
	**Del < 500kb**	1961	816	1.02 (0.94-1.11)	0.58	2389	2309	1.01 (0.96-1.07)	0.64
	**Dup < 500kb**	2515	1010	0.99 (0.92-1.06)	0.74	2744	2636	1.01 (0.96-1.06)	0.79
	**All ≥ 500kb**	506	226	**1.10 (0.94-1.29)**	0.24	447	493	**1.16 (1.02-1.32)**	**0.026**
	**Del ≥ 500kb**	124	63	1.25 (0.92-1.69)	0.15	91	114	**1.29 (0.99-1.69)**	0.059
	**Dup ≥ 500kb**	382	163	1.05 (0.87-1.26)	0.61	356	379	**1.12 (0.97-1.29)**	0.13
	**11 Loci**	105	56	**1.31 (0.95-1.80)**	0.10	40	47	**1.26 (0.83-1.92)**	0.28
MGS	**All < 500kb**	4549	2161	1.01 (0.96-1.06)	0.67	3193	3789	1.00 (0.96-1.05)	0.94
	**Del < 500kb**	2733	1274	0.99 (0.93-1.06)	0.84	1846	2214	1.01 (0.95-1.08)	0.69
	**Dup < 500kb**	1816	887	1.04 (0.96-1.12)	0.35	1347	1575	0.99 (0.92-1.06)	0.75
	**All ≥ 500kb**	269	154	**1.22 (1.00-1.49)**	**0.049**	152	253	**1.41 (1.15-1.72)**	**0.00093**
	**Del ≥ 500kb**	96	48	1.06 (0.76-1.48)	0.73	36	68	**1.58 (1.06-2.35)**	**0.026**
	**Dup ≥ 500kb**	173	106	**1.31 (1.03-1.67)**	**0.030**	116	185	**1.35 (1.07-1.71)**	**0.011**
	**11 Loci**	44	37	**1.72 (1.11-2.65)**	**0.015**	15	18	1.01 (0.51-2.02)	0.97
ISC	**All < 500kb**	4801	2687	1.04 (0.99-1.08)	0.11	3408	4341	1.00 (0.96-1.05)	0.96
	**Del < 500kb**	2431	1395	1.06 (1.00-1.13)	0.60	1749	2343	1.04 (0.98-1.11)	0.17
	**Dup < 500kb**	2370	1292	1.01 (0.95-1.08)	0.66	1659	1998	0.96 (0.90-1.02)	0.17
	**All ≥ 500kb**	320	173	1.04 (0.87-1.26)	0.67	175	215	0.99 (0.81-1.22)	0.96
	**Del ≥ 500kb**	104	56	1.05 (0.76-1.46)	0.76	29	56	**1.65 (1.04-2.60)**	**0.030**
	**Dup ≥ 500kb**	216	117	1.04 (0.83-1.30)	0.76	146	159	0.87 (0.70-1.09)	0.24
	**11 Loci**	51	31	**1.22 (0.77-1.92)**	0.40	12	11	0.79 (0.34-1.82)	0.56

§Control and case samples included in the test: 5,493 females and 5,762 males in CLOZUK controls, 1,938 females and 4,766 males in CLOZUK cases; 1,856 females and 1,567 males in MGS controls, 1,015 females and 2,162 males in MGS cases; 1,707 females and 1,478 males in ISC controls and 1,151 females and 2,244 males in ISC cases

*Odds ratios (and 95% confidence intervals) of females *vs* males in the number of CNVs they carry

^P-values of correlation of number of CNVs and gender generated from logistic regression models

All: deletions and duplications

Del: deletions

Dup: duplications

3. Gender burden of CNV gene content in combined dataset

Table S3. Gender burden of CNV gene content

CNV type	Cases				Controls			
	Male (N genes)	Female (N genes)	OR (95% CI)*	P-value^	Male (N genes)	Female (N genes)	OR (95% CI)*	P-value^
**Del ≥ 500kb**	4010	2399	1.01 (1.00-1.02)	0.082	1156	1535	1.01 (0.99-1.02)	0.29
**Dup ≥ 500kb**	6692	3602	1.01 (1.00-1.01)	0.12	4085	4897	1.01 (0.99-1.01)	0.22
**Del < 500kb**	5590	2667	1.00 (0.98-1.03)	0.71	4756	5469	1.01 (0.99-1.04)	0.18
**Dup < 500kb**	11000	5293	1.01 (0.99-1.02)	0.21	9643	9949	1.00 (0.99-1.01)	0.53

*Odds ratios (95% confidence intervals) of females *vs* males in the number of genes hit by CNVs
^P-values of correlation of number of gene hits and female generated from logistic regression model
Del: deletions
Dup: duplications

4. Gender stratification effect on schizophrenia CNV associations in the different datasets that comprise our main analysis

Table S4 presents a breakdown of the association of known specific schizophrenia observed in the different datasets that comprise our main analysis when gender was used as a stratification factor. This table is available as a separate Excel file.
